# Identification of the *Eutrema salsugineum EsMYB90* gene important for anthocyanin biosynthesis

**DOI:** 10.1186/s12870-020-02391-7

**Published:** 2020-04-28

**Authors:** Yuting Qi, Caihong Gu, Xingjun Wang, Shiqing Gao, Changsheng Li, Chuanzhi Zhao, Chuanshun Li, Changle Ma, Quan Zhang

**Affiliations:** 1 Shandong Provincial Key Laboratory of Plant Stress Research, College of Life Science, Shandong Normal University, Jinan, 250014 China; 2 Biotechnology Research Center, Shandong Academy of Agricultural Sciences, Shandong Provincial Key Laboratory of Crop Genetic Improvement, Ecology and Physiology, Jinan, 250100 China; 3 Beijing Engineering Research Center for Hybrid Wheat, The Municipal Key Laboratory of the Molecular Genetics of Hybrid Wheat, Beijing Academy of Agriculture and Forestry Sciences, Beijing, 100097 China

**Keywords:** Anthocyanin, Flavonoid, *Eutrema salsugineum*, R2R3 MYB transcription factor, *Es*MYB90, Transcriptional regulation, Anthocyanin biosynthesis genes

## Abstract

**Background:**

Anthocyanins contribute to coloration and antioxidation effects in different plant tissues. MYB transcription factors have been demonstrated to be a key regulator for anthocyanin synthesis in many plants. However, little information was available about the MYB genes in the halophyte species *Eutrema salsugineum*.

**Result:**

Here we report the identification of an important anthocyanin biosynthesis regulator *Es*MYB90 from *Eutrema salsugineum*, which is a halophyte tolerant to multiple abiotic stresses. Our phylogenetic and localization analyses supported that *Es*MYB90 is an R2R3 type of MYB transcription factor. Ectopic expression of *EsMYB90* in tobacco and *Arabidopsis* enhanced pigmentation and anthocyanin accumulation in various organs. The transcriptome analysis revealed that 42 genes upregulated by *Es*MYB90 in *35S*:*EsMYB90* tobacco transgenic plants are required for anthocyanin biosynthesis. Moreover, our qRT-PCR results showed that *Es*MYB90 promoted expression of early (*PAL*, *CHS*, and *CHI*) and late (*DFR*, *ANS*, and *UFGT*) anthocyanin biosynthesis genes in stems, leaves, and flowers of *35S*:*EsMYB90* tobacco transgenic plants.

**Conclusions:**

Our results indicated that *Es*MYB90 is a MYB transcription factor, which regulates anthocyanin biosynthesis genes to control anthocyanin biosynthesis. Our work provides a new tool to enhance anthocyanin production in various plants.

## Background

Flavonoids which are derivatives of the phenylpropanoid/flavonoid pathway mainly contain proanthocyanidins (PAs), anthocyanins and flavonols [1–3]. As important pigments, anthocyanins are responsible for red, purple, violet and blue colors in flowers, fruits, and leaves, which determine economic traits of crops and ornamental plants [4–7]. Anthocyanins are the end products of a specific branch in the phenylpropanoid/flavonoid biosynthesis pathway. Enzymes involved in anthocyanin biosynthesis have been extensively studied in many plant species [8]. Catalyzed by phenylalanine ammonia-lyase (PAL), the initial step of the flavonoid pathway is the conversion of phenylalanine into trans-cinnamic acid [9], while chalcone synthase (CHS) catalyzes the first committed step in the flavonoid biosynthesis to form naringenin chalcone. Chalcone isomerase (CHI) cyclizes chalcone to form naringenin [8]. The naringenin is then converted into dihydrokaempferol (DHK) by flavanone 3 β-hydroxylase (F3H). DHK is further hydroxylated to dihydroquercetin (DHQ) by flavonoid 3’-hydroxylase (F3’H), or to dihydromyricetin (DHM) by flavonoid 3’,5’-hydroxylase (F3’5’H). Dihydroflavonol 4-reductase (DFR) converts DHQ into leucocyanidin, which is further converted into anthocyanidins by anthocyanidin synthase (ANS). Finally, UDP-glucose: flavonoid 3-O-glucosyltranferase (UFGT) catalyzes glycosylation of anthocyanidins to form anthocyanins [8, 10–12].

MYB transcription factors play a central role in regulating expression of genes encoding major enzymes for anthocyanin biosynthesis via forming the transcriptional complex containing MYB-bHLH-WD40 (MBW) [1, 13–15]. Expression of early biosynthesis genes (EBGs) such as *CHS* and *CHI*, is regulated by MYB11, MYB12 and MYB111, whereas PAP1 to PAP4 (*At*Myb75, *At*Myb90, *At*Myb113, and *At*Myb114) control expression of late biosynthesis genes (LBGs) including *DFR*, *ANS*, and *UFGT* in *Arabidopsis* [16, 17]. In *Arabidopsis*, up-regulation of one of *MYB75*, *MYB90*, *MYB113* and *MYB114* genes is sufficient to increase anthocyanin accumulation in young leaves [15, 17]. For example, the well-known *Arabidopsis AtMYB75* (*PAP1*) gene directs anthocyanin production in leaves, roots, flowers, and fruits [18, 19]. Overexpression of *AtMyb75* in *Arabidopsis* and tobacco results in upregulation of *PAL*, *CHS* and *DFR* genes [19, 20]. Similarly, *At*MYB75 induces anthocyanin production in tomato (*Solanum lycopersicum* L.) via promoting the *DFR* expression [18]. Furthermore, the sequence variation of *AtMYB90* (*PAP2*) is causal for natural variation in anthocyanin accumulation [17, 21]. *At*MYB90 may act together with TTG1 (a WD40 protein) and different bHLH partners including TT8, GL3 or EGL3 [15, 18, 22]. Moreover, in *Arabidopsis* the ternary complexes formed by R2R3-MYB, bHLH and the WD repeat protein activate the biosynthetic genes required for proanthocyanidin accumulation in the innermost cell layer of the seed coat [23]. The R2R3 MYB protein TT2 (MYB123) is also a key regulator of proanthocyanidin accumulation in developing seeds [24].

MYB transcription factors are involved in regulation of anthocyanin synthesis in many plants, such as *Arabidopsis* [15, 17, 24, 25], cauliflower (*Brassica oleracea var botrytis*) [26], bok choy (*Brassica rapa var. chinensis*) [27], apple (*Malus × domestica*) [11, 28–30], peach (*Prunus persica*) [14, 31], pear (*Pyrus pyrifolia*) [13, 32, 33],strawberry (*Fragaria x ananassa*) [34], snapdragon (*Antirrhinum majus*) [35], *Chrysanthemum* [10], grape hyacinth (*Muscari armeniacum*) [36], grapevine (*Vitis vinifera*) [37, 38], chinese bayberry (*Myrica rubra*) [6, 39], *Epimedium sagittatum* [40, 41], poplar (*Populus spp*) [42] and potato (*Solanum tuberosum L*) [43]. In addition, some *MYB* genes are up-regulated under various stress conditions [15, 17]. However, the transcriptional regulation of anthocyanin synthesis by MYB in stress-tolerant plants is not well studied. *Eutrema salsugineum* (salt cress), a stress-tolerant model halophyte, is highly tolerant to cold, salt, drought, oxidative stress, and nitrogen deficiency. In *Eutrema salsugineum*, many stress-tolerant related genes, such as *SOS1*, *HKT1,* and *nsLTP4*, have been identified [44–49]. By analyzing the differentially expressed regulatory genes between *Arabidopsis* and *E.salsugineum*, it was found that the regulatory functions of 307 transcription factors in 50 different families were significantly different [50]. Another study found that *EsMYB96/WAX1* from *E.salsugineum* under the *RD29A* promoter improved drought tolerance with increased accumulation of cuticular wax and ascorbic acid in transgenic *Arabidopsis* [51]. So far, there is no research report on the anthocyanin synthesis of *EsMYBs* in *E.salsugineum*. Here, we reported our functional analysis of the MYB transcription factor *Es*MYB90 in anthocyanin synthesis. Our phylogenetic and localization analyses suggest that *Es*MYB90 is an R2R3 type of MYB transcriptional factor. Ectopic expression of *EsMYB90* in tobacco and *Arabidopsis* led to significantly increased pigmentation and production of anthocyanins in leaves, stems, and flowers. Our further RNA-seq and qRT-PCR analyses showed that *Es*MYB90 promoted expression of anthocyanin early biosynthesis genes (EBGs: *NtCHS*, *NtCHI*, and *NtF3H*) and late biosynthesis genes (LBGs: *NtDFR*, *NtANS*, and *NtUFGT*) in 35S:*EsMYB90* tobacco transgenic plants. Our study identified a MYB transcription factor, which plays an important role in plant anthocyanin biosynthesis.

## Results

### Database mining identifies *Es*MYB90, a candidate regulator for anthocyanin synthesis

*Eutrema salsugineum* is a stress-tolerance halophyte, which produces purple flower buds after vernalization [52]. Since *MYB* genes are required for anthocyanin synthesis [13, 15, 36], we identified which *MYB* controls this purple phenotype in *E. salsugineum*. After comparing *MYB* genes obtained from the transcriptome of *E. salsugineum* based on our previously published results [46], with 72 *MYB* genes known acting as proanthocyanin (PA) and anthocyanin regulators in other plants, we found one candidate *MYB* gene, named as *EsMYB90*.

**Fig. 1:**
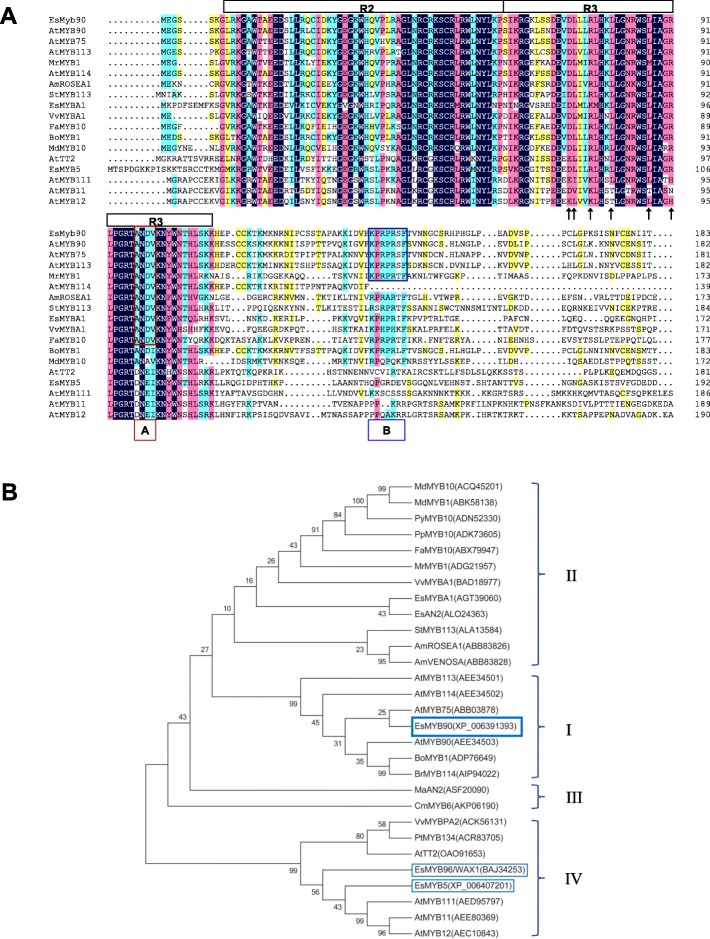
Alignment and phylogenetic analysis of *Es*MYB90 and other MYB proteins. **a** The *Es*MYB90 protein sequence was aligned with a set of related R2R3-MYB proteins from 10 plant species. Identical amino acids are shaded in dark blue, and the greater than or equal to the identity of 75, 50, and 33% are indicated in shade of pink, light blue, and yellow, respectively. R2 and R3 domains refer to two repeats of the MYB DNA binding domain. Box (A): the conserved motif ANDV in the R3 domain for dicot anthocyanin-promoting MYBs; Box (B): the C-terminal-conserved motif KPRPR [S/T] F for *Arabidopsis* anthocyanin-promoting MYBs; Black arrows: the specific residues of [D/E]Lx_2_[R/K]x_3_Lx_6_Lx_3_R that confer to the interaction with bHLH. **b** Phylogenetic analysis of *Es*MYB90 and other 28 MYB proteins. The MYB protein sequences were downloaded from the GenBank database with accession numbers showed in the diagram. *Es*MYB5, *Es*MYB90 and *Es*MYB96 are from *E. salsugineum*, and *Es*MYB90 is highlighted in a bold blue box, while *Es*MYB5 and *Es*MYB96 are highlighted in a thin blue box. MYB proteins from the *cruciferae* plants in the clade I have higher identities to *Es*MYB90 than that in clades II to IV

To determine the relationship of *Es*M*YB*90 to characterized flavonoid and PA MYBs, we performed similarity analysis at the protein level. Our results showed that *Es*MYB90 has 80.5, 78.9, 78.4, 74.4, 69.4, 65.9, 50% identities respectively to 7 MYB proteins, i.e. *Bo*MYB1, *At*MYB90, *Br*MYB114, *At*MYB75, *Cr*MYB114, *At*MYB113, and *At*MYB114 (Fig. 1a). In addition, similarities between *Es*MYB90 and other 10 MYB proteins range from 44.1 to 39.0% (Fig. 1a). Those MYB proteins with high similarities to *Es*MYB90 belong to the class of R2R3-MYB, which have a conserved DNA-binding domain (R2 and R3 repeats) in the N-terminal and a variable C-terminal region [41, 53, 54]. The ANDV motif (marked by red A box in Fig. 1a), a characteristic identifier for anthocyanin-promoting MYBs in dicots [10], existed in *Es*MYB90, *At*MYB90, *At*MYB75, *At*MYB113, *At*MYB114, *Am*ROSEA1, *St*MYB113, *Es*MYBA1(AGT39060), *Vv*MYBA1, *Mr*MYB1, and *Fa*MYB10, while the C-terminal-conserved motif KPRPR [S/T] F for *Arabidopsis* anthocyanin-promoting MYBs [25, 36] (marked by blue B box in Fig. 1a), was only found in *Es*MYB90, *At*MYB75, *At*MYB90, *At*MYB113, and *Mr*MYB1. Moreover, *Es*Myb90 has a conserved [D/E]Lx2[R/K]x3Lx6Lx3R motif (marked by black arrows in Fig. 1a), which is required for interaction with R/B-like bHLH proteins [10, 16].

To further identify the relationship of *Es*MYB90 to other MYB proteins, we generated a phylogenetic tree with 29 MYB proteins involved in anthocyanin synthesis in 16 plants. Our results demonstrated that *Es*MYB90 was clustered in the clade I (Fig. 1b), which consists of *At*MYB75, *At*MYB90, *At*MYB113, *At*MYB114, *Bo*MYB1 and *Br*MYB114 that are important for anthocyanin accumulation [17, 19, 25–27, 55]. *Es*MYB90 has a relatively farer phylogenetic relationship to MYB proteins in clades II, III, and IV, although those MYBs promote biosynthesis of PA and anthocyanin, except for *Es*MYB5(XP_006407201) have no research reports yet.

In summary, our results suggest that *Es*MYB90 is a R2R3-MYB, which may function in proanthocyanin and anthocyanin synthesis.

### Expression pattern of *Es*MYB90 in *E.salsugineum* and subcellular localization of the protein

**Fig. 2:**
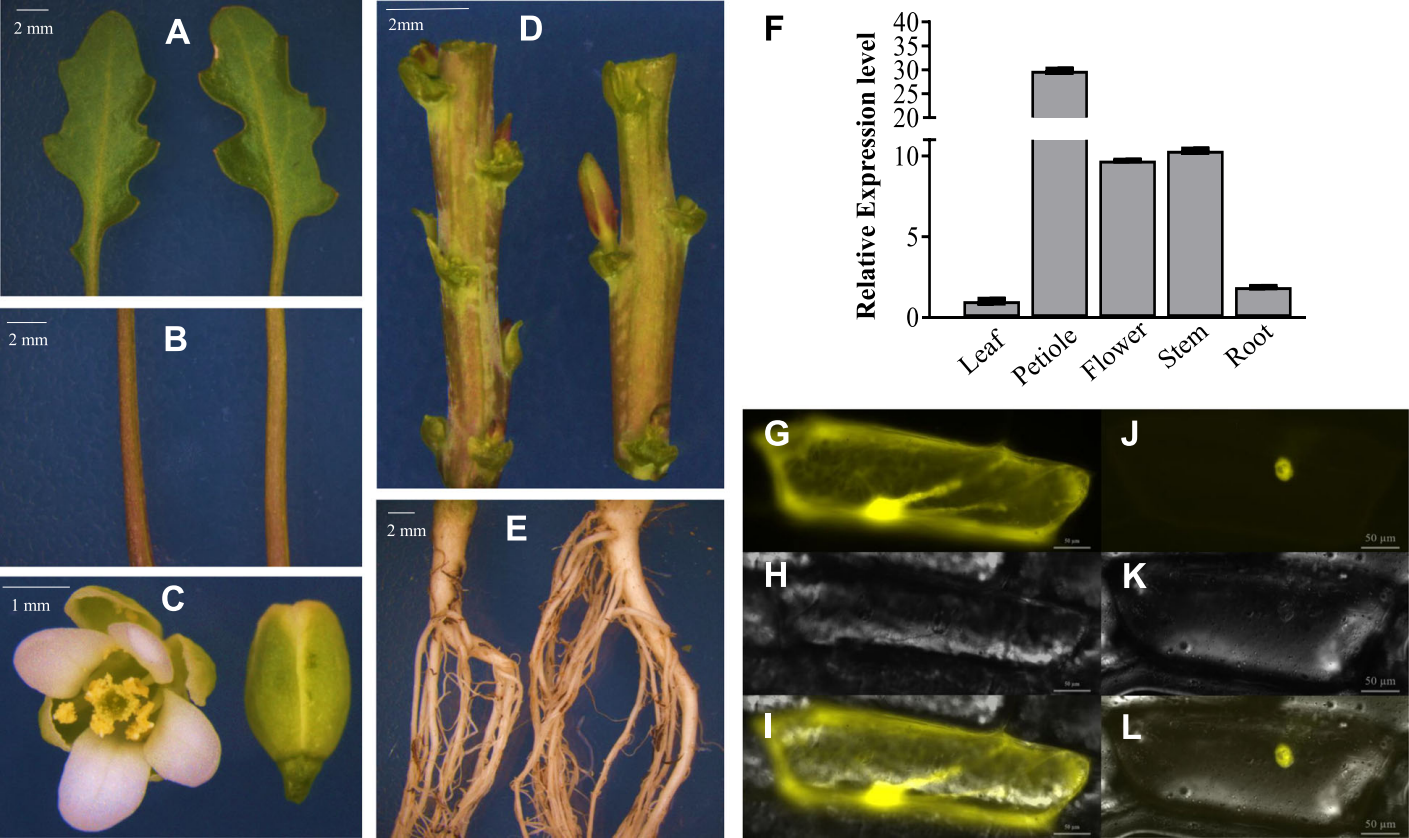
Expression pattern of *Es*MYB90 in *E. salsugineum* and subcellular localization of the protein. The leaves (**a**), petioles (**b**), flowers (**c**), stems (**d**), and roots (**e**) from thirty-five-week-old *E.salsugineum* plants were collected for phenotypic observation. **f** The expression levels of *EsMyb90* in leaves, petioles, flowers, stems, and roots from thirty-five-week-old *E. salsugineum*, and vertical bars indicate standard errors of the 3 biological replicates. Transient expression of the 35S:*YFP*-*EsMYB90* in onion epidermal cells showing *EsMYB90* is localized in the nucleus. **g** An onion epidermal cell expressing *35S:YFP* showing YFP signals in both cytoplasm and nucleus; (**h**) An onion epidermal cell expressing *35S:YFP* in the bright field; (**i**) The merged image of G and H; (**j**) An onion epidermal cell expressing *35S:YFP-EsMYB90* exhibiting the YFP signal only in the nucleus; (**k**) An onion epidermal cell expressing *35S:YFP-EsMYB90* in the bright field; (**l**) The merged image of J and K. The control was *35S:YFP*. Bars: 50 μm

In order to detect the expression pattern of *EsMYB90*, we collected leaves, petioles, stems, roots and flowers of *E.salsugineum*, and performed qRT-PCR. Our result showed that *EsMYB90* was expressed in all examined tissues of *E.salsugineum*, among which it has the highest expression level in petiole (Fig. 2b), followed by stems and flowers (Fig. 2c,d), but a relative lower expression in leaves and roots (Fig. 2a,e). This result is consistent with the color phenotype of different tissues observed, suggesting the expression of *EsMYB90* is related to the synthesis of anthocyanins (Fig. 2a-f).

To test the subcellular localization of *Es*MYB90, we examined the transient expression of YFP-*Es*MYB90 fusion protein in onion epidermal cells. Our results showed that YFP signals were observed in both cytoplasm and nucleus of the onion epidermal cells expressing *35S:YFP* (Fig. 2g-i), while the YFP signal was only detected in the nucleus in cells expressing *35S:YFP-EsMYB90* (Fig. 2j-l). Our result showed *Es*MYB90 is localized to the nucleus, suggesting that as other MYB proteins *Es*MYB90 also functions as a transcriptional regulator.

### Ectopic expression of *EsMYB90* promotes anthocyanin accumulation in tobacco and *Arabidopsis*

To investigate the possible function of *EsMYB90* in anthocyanin biosynthesis, we generated the *35S:EsMYB90* construct to ectopically expression *EsMYB90* in tobacco and *Arabidopsis* (Additional file 1: Fig. 1a). Eighteen *35S*:*EsMYB90* transgenic tobacco and 15 *35S*:*EsMYB90* transgenic *Arabidopsis* plants were obtained, respectively (Additional file 1: Fig. 1b,c).

**Fig. 3:**
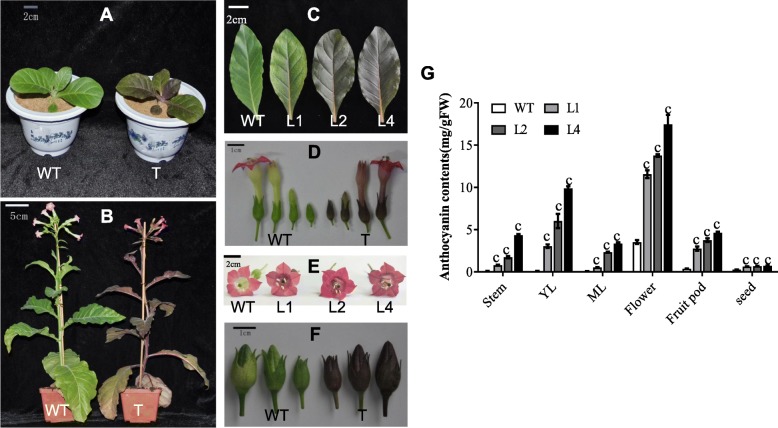
Ectopic expression of *EsMYB90* increases anthocyanin contents in tobacco. **a** Five-week-old wild-type (WT) and tobacco transgenic (T) seedlings; (**b**) Nine-week-old WT and tobacco T plants at the flowering stage; (**c**) Leaves from six-week-old WT as well as L1, L2 and L4 tobacco transgenic plants; (**d**) Sepal and corolla from nine-week-old WT and tobacco transgenic plants; (**e**) Flowers from nine-week-old WT as well as L1, L2 and L4 tobacco transgenic plants; (**f**) Fruit pods from nine-week-old WT and tobacco transgenic plants; (**g**) Anthocyanin contents in stems, young leaves (YL), mature leaves (ML), flowers, fruit pods of eight-week-old plants, and mature seeds from WT as well as L1, L2 and L4 tobacco transgenic plants. Vertical bars indicate standard errors of 3 biological replicates and the Student’s t test values are indicated as c (*P* < 0.001)

We found that in all developing stages, leaves and stems in *35S*:*EsMYB90* tobacco plants appeared purple-red, and the color became deepened with development (Fig. 3a-c). In addition, *35S*:*EsMYB90* tobacco plants produced purple-red corollas, purple-black sepals, and purple-black fruit pods, whereas wild-type corollas were pink, with green sepals and fruit pods (Fig. 3d-f). Our results from examining anthocyanin production showed that the total anthocyanin contents in three *35S*:*EsMYB90* tobacco lines were significantly increased in stems, young leaves (YL), mature leaves (ML), flowers, fruit pods, and mature seeds, compared with the wild type (Fig. 3g). Among L1, L2 and L4 three tested lines, the L4 transgenic line had the highest anthocyanin contents. Compared with the wild type, the total anthocyanin contents in young leaves (YL), mature leaves (ML), stems, flowers, fruits pods, and mature seeds of the L4 line were increased 95.2, 45.7, 48.8, 4.9, 17.8, and 2.6 folds, respectively (Fig. 3g). These results indicate that the enhanced pigmentation in *35S*:*EsMYB90* tobacco plants was caused by the increased synthesis of anthocyanins.

**Fig. 4:**
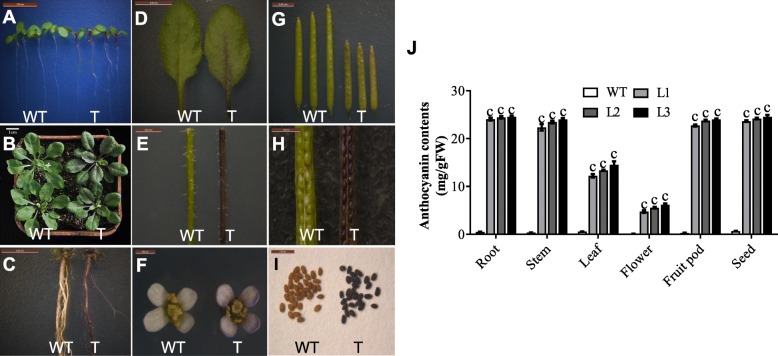
Ectopic expression of *EsMYB90* increases anthocyanin contents in *Arabidopsis*. (**a**) Seven-day-old wild-type (WT) and *Arabidopsis* transgenic (T) seedlings; (**b**) Three-week-old WT and *Arabidopsis* T plants; (**c**) Roots from seven-week-old WT and *Arabidopsis* T plants; (**d**) Leaves from three-week-old WT and *Arabidopsis* T plants; (**e**) Stems from seven-week-old WT and *Arabidopsis* T plants; (**f**) Flowers from seven-week-old WT and *Arabidopsis* T plants; (**g**) and (**h**) Fruit pods from seven-week-old WT and *Arabidopsis* T plants; (**i**) Seeds from nine-week-old WT and *Arabidopsis* T plants; (**j**) Anthocyanin contents in roots, stems, leaves, flowers, fruit pods of four-week-old plants and mature seeds, from WT as well as L1, L2 and L3 *Arabidopsis* transgenic plants. Vertical bars indicate standard errors of the 3 biological replicates and the Student’s t test values are indicated as c (*p* < 0.001)

We observed similar phenotypes in *35S*:*EsMYB90 Arabidopsis* transgenic plants. In comparison to the wild-type plants, the color of leaves, roots, stems, flowers, fruit pods, and seeds became light-purple to dark-purple in *35S*:*EsMYB90 Arabidopsis* plants (Fig. 4a-g). In particularly, seeds from *35S*:*EsMYB90 Arabidopsis* plants exhibited black color (Fig. 4h,i). Furthermore, the contents of anthocyanins in the roots, stems, leaves, flowers, and fruit pods at the bolting stage, and the mature seeds from three *35S*:*EsMYB90 Arabidopsis* transgenic lines (L1, L2, and L3) were significantly higher than that in wild-type plants (Fig. 4j).

Collectively, our results suggest that *Es*MYB90 functions as a transcription factor to promote anthocyanin biosynthesis in plants.

### Transcriptomic analyses show that *Es*MYB90 is a key regulator in the proanthocyanidin and anthocyanin pathway

**Fig. 5:**
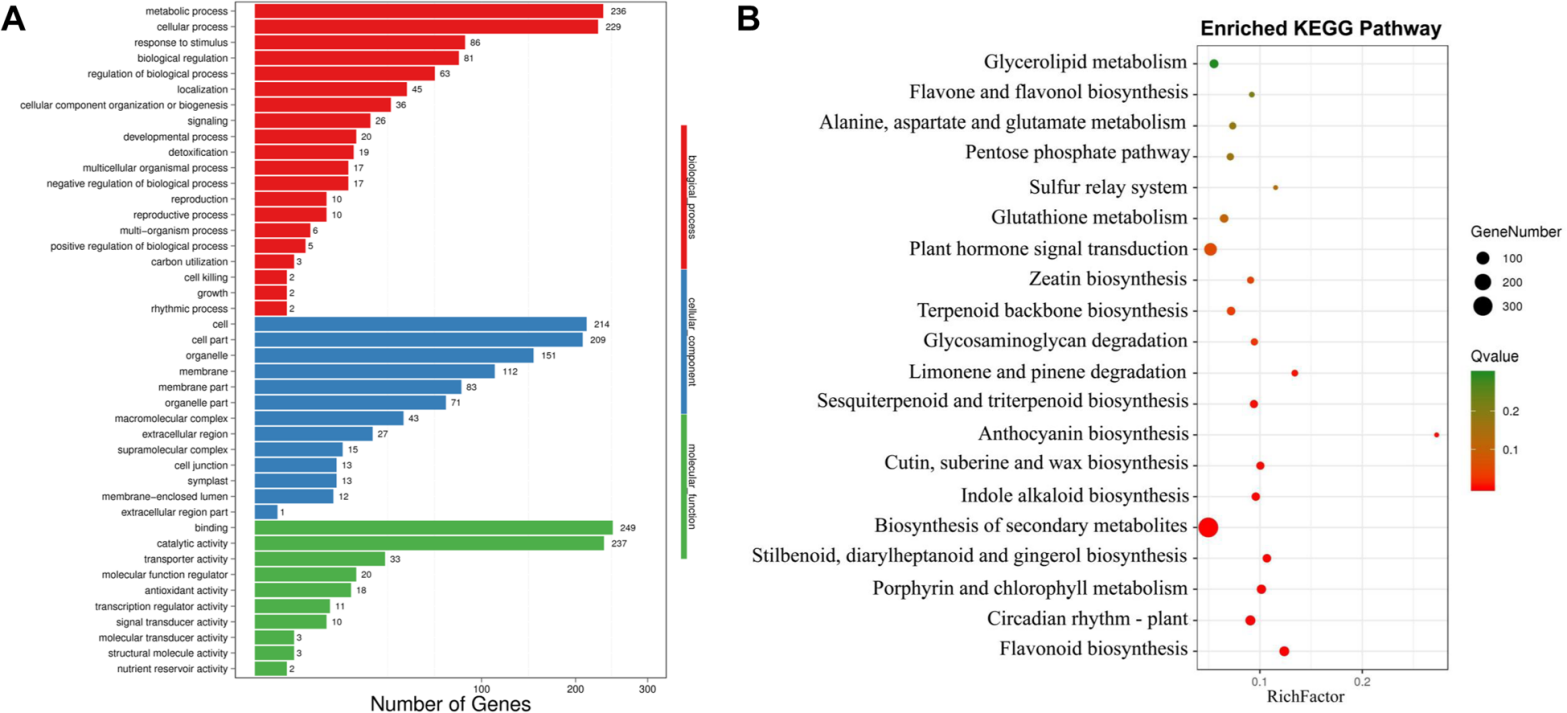
Analysis of GO terms and KEGG-enriched differentially expressed genes (DEGs). **a** The annotated GO terms in DEGs. The X axis represents the number of DEGs. The Y axis indicates the classification of GO terms. **b** The top 20 pathways of enriched KEGG DEGs. The X axis represents the enrichment factor value, while the Y axis indicates the pathway names. The color of dots represents the q value. The size of dots represents the number of DEGs. The rich factor refers to the value of enrichment factor

To examine the molecular mechanisms by which *Es*MYB90 controls anthocyanin biosynthesis in the genome wide, we performed RNA-seq analysis using the leaves from wild-type and *35S*:*EsMYB90* tobacco transgenic plants. We identified 51,202 differentially expressed genes (DEGs) in the comparison of wild-type plant with *35S*:*EsMYB90* transgenic tobacco plants, among which 2446 DEGs have log2 Fold Change ≥1 or ≦ − 1 and a Padj ≤0.05 (Additional file 2). Furthermore, 1199 out of 2446 DEGs were up-regulated, while 1247 genes were down-regulated (Additional file 2). Moreover, 476 unique DEGs were annotated into 43 GO terms, wherein the GO terms with the top 3 of the number of DEGs encoding the binding (249 genes), catalytic activities (237 genes) and metabolic processes (236 genes) (Fig. 5a, Additional file 3).

We revealed that among 2446 DEGs, 1023 unique genes were annotated into the 128 KEGG pathways (Additional file 4). The most prominent KEGG-enriched genes are involved in secondary metabolite biosyntheses, followed by plant hormone signaling, flavonoid biosynthesis (Additional file 1: Fig. 2). According to the q value of DEGs, the top 20 of enrichment paths included the flavonoid biosynthesis (ko00941), anthocyanin biosynthesis (ko00942), flavone and flavonol biosynthesis (ko00944), plant circadian rhythm (ko04712),and glutathione metabolism (ko00480;Fig. 5b, Additional file 5). Moreover, the anthocyanin biosynthesis pathway has the largest enrichment factor, followed by the pathway of flavonoid biosynthesis. The flavone and flavonol biosynthesis pathways also has a large enrichment factor (Fig. 5b, Additional file 5).

**Fig. 6:**
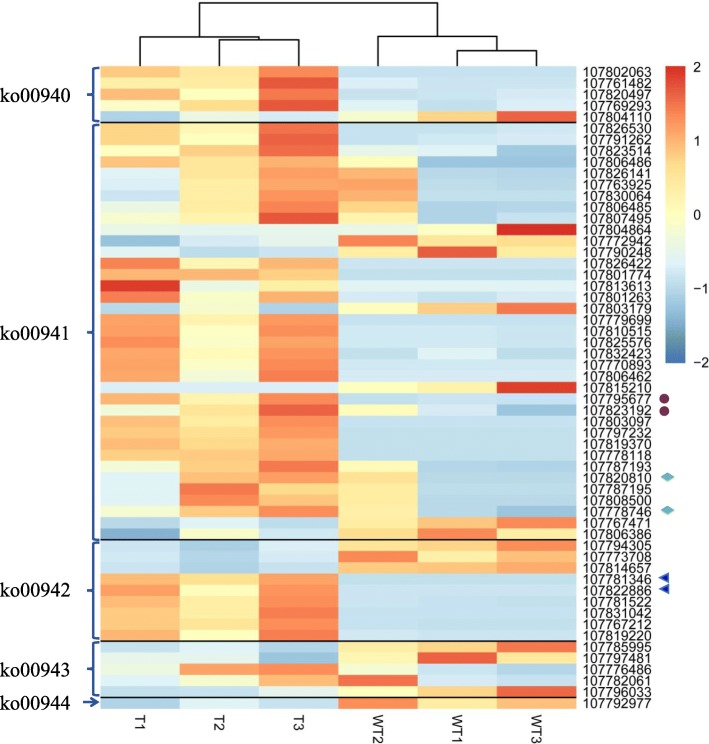
Heatmap showing transcription levels of DEGs in flavonoid and anthocyanin synthesis pathways (ko00940-ko00944). Purple solid circles indicate 107,795,677 and 107,823,192 genes, which also exist in the ko00944 pathway. Green diamonds indicate 107,820,810 and 107,778,746 genes, which can be also found in the ko00942 pathway. Blue triangles indicate 107,781,346 and 107,822,886 genes, which co-exist in the ko00944 pathway

Mapping to the KEGG reference pathways found that a total of 57 significantly differential expression genes were assigned to five secondary metabolic pathways, i.e. phenylpropanoid biosynthesis (ko00940), flavonoid biosynthesis (ko00941), anthocyanin biosynthesis (ko00942), isoflavonoid biosynthesis (ko00943), and flavone and flavonol biosynthesis pathways (ko00944). The gene names, gene ID, and the combined functional annotations were seen in Additional file 6. Out of 57 genes, 42 genes encode PA and anthocyanin biosynthesis enzymes, such as PAL (107,802,063, 107,761,482 and 107,820,497), CHS (107,826,422, 107,801,774 and 107,813,613), CHI (107,779,699, 107,810,515 and 107,825,576), F3H (107,770,893, 107,806,462), DFR (107,803,097,107,797,232), and ANS/ LDOX (107,819,370, 107,778,118, 107,787,193, 107,787,195 and 107,808,500). Particularly, six genes encoding UFGT (107,781,346, 107,822,886, 107,781,522, 107,831,042, 107,767,212 and 107,819,220) in the anthocyanin biosynthesis pathway (ko00942) were all strongly up-regulated (Fig. 6, Additional file 6). However, in ko00940-ko00944 pathways, only 15 genes including that encoding flavonol synthase/flavanone 3-hydroxylase (107,794,305, 107,814,657), trans-resveratrol di-O-methyltransferase-like (107,785,995, 107,797,481), and flavone 3′-O-methyltransferase 1-like (107792977), were down-regulated (Fig. 6, Additional file 6).

Taken together, our RNA-seq results demonstrated that identified DEGs are significantly enriched in the flavonoid and anthocyanin synthesis pathway (ko00941-ko00944), suggesting that *Es*MYB90 play an important regulatory role in proanthocyanidin and anthocyanin synthesis.

### Validation of RNA-seq results by qRT-PCR

**Fig. 7:**
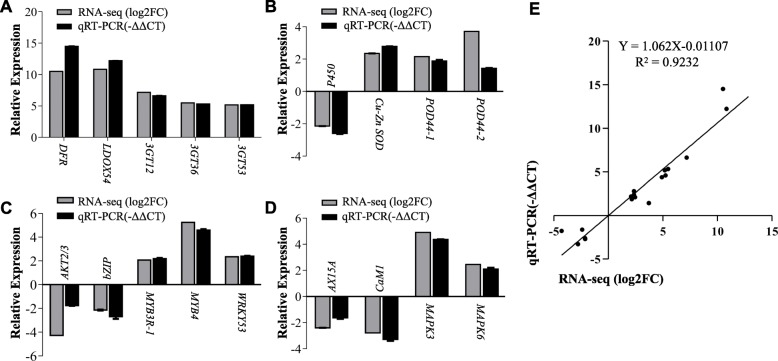
A-D, Validation of RNA-seq results using quantitative real-time PCR. (**a**) Anthocyanin biosynthesis genes; (**b**) Antioxidant related genes; (**c**) Genes encoding transcription factor and ion channel; (**d**) Signaling pathway related genes. *Ntubi2* (*Ntubiquitin 2*, LOC107772211) was used as an internal control. E, Correlation analysis of RNA-Seq and qRT-PCR results. The correlation of the fold change analyzed by RNA-Seq (x-axis) with data obtained using qRT-PCR (y-axis). Each set of data were obtained from three repeats

To validate the RNA-seq results, we performed quantitative reverse transcription PCR (qRT-PCR) for 18 genes which are assigned to 5 groups related to anthocyanin biosynthesis, antioxidant production, signal transduction, transcription regulation, and ion channel in tobacco (Additional file 7). Our results showed that expression level changes of 5 anthocyanin biosynthesis genes [*NtDFR* (107803097), *NtLDOX54* (107778118), *Nt3GT12* (107781346), *Nt3GT36* (107781522), and *Nt3GT53* (107831042)] detected by qRT-PCR were in agreement with the RNA-seq data (Fig. 7a). We obtained similar qRT-PCR results from examining expression of 4 antioxidant-related genes [*NtP450* (107772738), *NtCu-ZnSOD* (107806960), *NtPOD44–1* (107827231), and *NtPOD44–2* (107797651); Fig. 7b], 4 genes encoding transcription factors [*NtbZIP* (107795590), *NtMYB3R-1* (107795213), *NtMYB4* (107802984), and *NtWRKY53* (107825953); Fig. 7c], *NtAKT2/3*(107761230) encoding a potassium channel protein (Fig. 7C), and 4 genes related to signal transduction and ion channel [*NtMAPK3* (107782983), *NtMAPK6* (107806359), *NtAX15A* (107805986), and *NtCaM1* (107803626); Fig. 7d]. We found similar differential expression patterns for the DEGs in the qRT-PCR and RNA-seq data, with a lower pearson’s coefficient (R2) as 0.9232. Therefore, qRT-PCR results support that our transcriptome results are reliable.

### *EsMYB90* promotes expression of anthocyanin biosynthetic genes in tobacco

To further elucidate the molecular function of *Es*MYB90 in proanthocyanin and anthocyanin biosynthesis, we examined expression of key anthocyanin biosynthesis genes *PAL*, *CHS*, *CHI*, *F3H*, *F3’H*, *DFR*, *ANS*/*LDOX*, and *UFGT* in the stems, young leaves (YL) and flowers from *35S:EsMYB90* tobacco transgenic lines (L2, L4) and wild-type tobacco plants at the flowering stage by qRT-PCR.

**Fig. 8:**
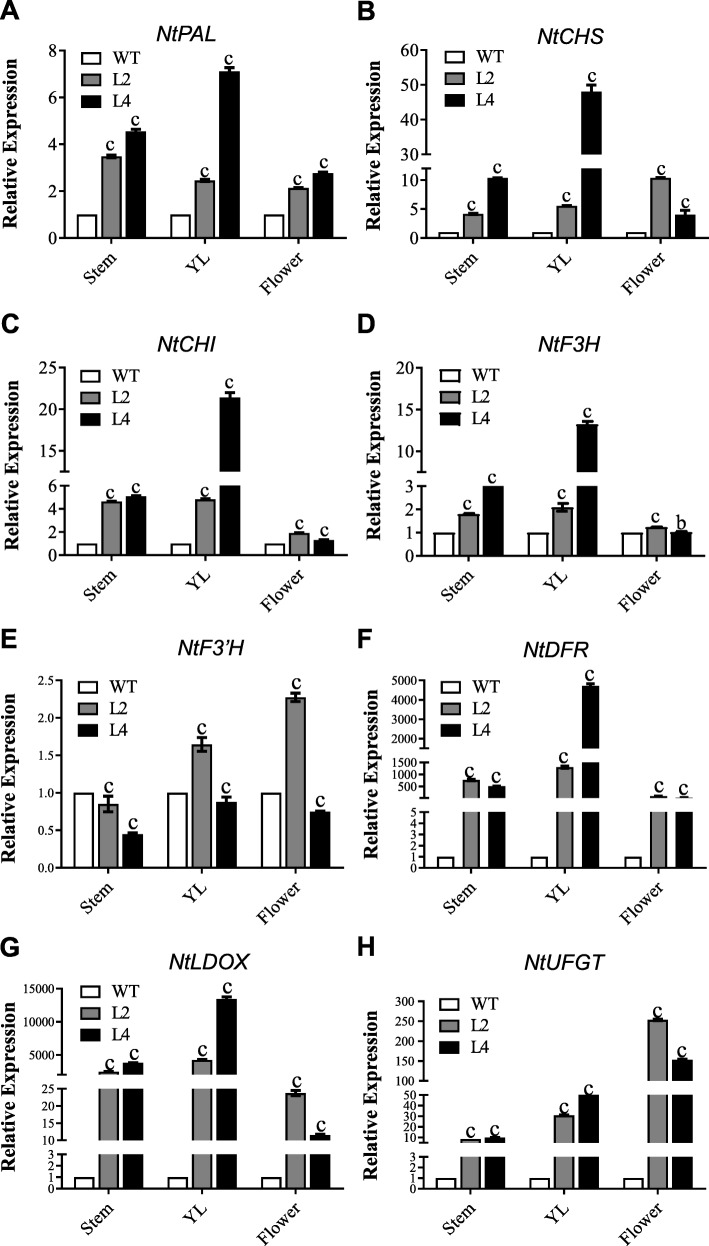
Expression analysis of anthocyanin biosynthesis genes in stem, leaf and flower from two *EsMYB90* transgenic tobacco lines at the flowering stage. WT: Wild Type; L2 and L4: two independent *35S:EsMYB90* lines*. Ntubi2* (*Ntubiquitin 2*, LOC107772211) was used as an internal control. (**a**) *NtPAL* (XM_016625506.1); (**b**) *NtCHS* (NM_001326166.1); (**c**) *NtCHI* (NM_001325287.1); (**d**) *NtF3H* (NM_001325083.1); (**e**) *NtF3’H* (NM_001325608.1); (**f**) *NtDFR* (NM_001325732.1); (**g**) *NtLDOX* (NM_001326043.1); (**h**) *NtUFGT* (NM_001325312.1). The ΔΔCt method was used to determine the relative expression, and expression levels of tested genes in the wild type were set to 1. Vertical bars represent standard errors of three biological replicates. b and c indicate p < 0.01 and p < 0.001, respectively

PAL is the first key enzyme in the metabolic pathway of phenylpropanoid [9]. Expression levels of *NtPAL* in stems, leaves and flowers from the L4 line increased 4.6, 7.1, and 2.8 times, respectively, than that of wild type (Fig. 8a). CHS catalyzes the first step of anthocyanin biosynthesis, while CHI catalyzes the cyclization of chalcone molecules to form naringenin [8]. Expression levels of both *NtCHS* and *NtCHI* in stems, leaves and flowers from the L4 line were significantly increased compared to that in the wild type (Fig. 8b,c). Whereas, the relative transcript level of NtF3H in the flowers was slightly down-regulated in L4 transgenic line (Fig. 8d), and NtF3’H transcripts in the stems were down-regulated in L2 and L4 transgenic lines (Fig. 8e). Finally, the anthocyanin biosynthesis genes *NtDFR*, *NtANS* and *NtUFGT* which are required for anthocyanin biosynthesis at later steps were also significantly upregulated by *EsMYB90* (Fig. 8f-h).

In summary, our results suggest that *Es*MYB90 controls anthocyanin biosynthesis by promoting expression of anthocyanin biosynthesis genes, particularly LBGs.

## Discussion

Anthocyanins are main contributors to the coloration of plants, and the color is an important determinant for fruit and flower quality [8, 14, 56]. Flavonoids such as anthocyanins are also capable of scavenging oxygen free radicals produced in cells, thus have antioxidant activity [57, 58]. The antioxidant activity of anthocyanins depends on the degree of B-ring hydroxylation, acylation, and glycosylation [56]. Anthocyanins, as an antioxidant, can reduce the peroxidation of lipids and delay the aging of cells. Meanwhile, anthocyanins protect plants from damage caused by biotic and abiotic stresses and allow plants to adapt to environmental changes [59]. In higher plants, PAs and anthocyanin biosynthesis are regulated by different sets of MYB-bHLH-WD40 (MBW) complexes, and the R2R3-MYBs play vital roles in transcriptional regulation of anthocyanins biosynthesis [12, 19, 20, 23, 25, 34, 35]. Our results shed light on the molecular mechanism by which a novel R2R3-MYB controls PA and anthocyanin biosynthesis via promoting expression of PA and anthocyanin biosynthesis genes.

In *Arabidopsis*, MYB75, MYB90, MYB113, and MYB114 function redundantly by participating in the MBW (MYB-bHLH-WD40) complex to regulate PA and anthocyanin biosynthesis. Overexpression of one of these four MYBs is sufficient to increase anthocyanin accumulation in young leaves [15, 17]. *Es*MYB90 is a R2R3-MYB, containing a conserved [D/E]Lx2[R/K]x3Lx6Lx3R motif (black arrows) required for interaction with R/B-like bHLH proteins, which was not found in *Es*MYB15 (XP_006406061), *Es*MYB106(XP_006390428), *Es*MYB108 (XP_006407939), *Es*MYB -related protein 340 (XP_006395259) from *E. salsugineum*. The ANDV (red box A) and KPRPR [S/T] F motifs (blue box B) characterizing anthocyanin-promoting MYBs exist in *Es*MYB90 protein but not in other five *Es*MYBs [MYB5 (XP_006407201), MYB15, MYB106, MYB108, MYB-related Protein 340] (Additional file 1: Fig. 3). In *Arabidopsis* and some other higher plants, the anthocyanin pathway is controlled by multiple MYB transcriptional factors [1]. For instance, at least four MBW complexes assembled with various MYBs are involved in the PA accumulation in the innermost cell layer of *Arabidopsis* seed coat [23]. Thus, our results suggest that different from other plants less MYBs regulate anthocyanin biosynthesis in *E. salsugineum* and *Es*MYB90 is a major player.

The regulatory mechanisms for flavonoid biosynthesis are conserved in higher plants [1]. The MYB proteins usually determine the involvement of MBW complexes in specific pathways [1, 60]. In the MBW complex, MYBs have the highest binding specificity compared with bHLH and WD40. MYB and bHLH bind to at least one of three conserved *cis* elements, i.e. MYB-core, AC-rich, and E/G-box, in promoters of four LBGs (late biosynthesis genes: DFR, TT19, TT12, and AHA10), which specify their expression in the seed coat of *Arabidopsis* [23, 60]. Expression of LBGs are regulated by MYB75, MYB90, MYB113 and MYB114 in *Arabidopsis* [15, 16]. Our phylogenetic analysis showed that *Es*MYB90 was in the same clade with *Arabidopsis* MYB75, MYB90, MYB113, and MYB114, suggesting that *Es*MYB90 may play a more important role in regulation of LBGs (*DFR*, *ANS*/*LDOX* and *UFGT*) expressions, which is in accordance with our research results. In addition, our results show that *Es*MYB90 promotes expression of early biosynthesis genes, such as PAL, CHS, CHI, and F3H which agrees with findiCngs in other plants [1, 10, 36]. Furthermore, it is possible that *Es*MYB90 upregulates expressions of *NtMYB* and *NtbHLH* genes involved in anthocyanin biosynthesis, because expressions of *NtMYB3* (107820930), *NtMYB4* (107,802,984, 107,769,018, 107,815,562, 107,760,435), *NtMYB44* (107,816,351, 107,759,750), *MYB-like* (107,807,565, 107,795,213), *NtbHLH* (107,791,671, 107,791,200), *NtbHLH92* (107785534), *NtbHLH18* (107811232), and *NtbHLH35*(107774314) were significantly increased in *35S:EsMYB90* tobacco transgenic plants (Additional file 2).

Overall, our results showed that ectopic expression of the novel *EsMYB90* gene can strongly induce the anthocyanin biosynthesis by promoting expression of anthocyanin biosynthesis pathway genes, particularly functioning in the LBGs. Our study also paved the way for the application of *EsMYB90* to genetically engineering crops and ornamental plants.

## Conclusions

*EsMYB90*, a R2R3 MYB transcription factor, is localized in nucleus. *35S:EsMYB90* transgenic tobacco and *Arabidopsis* showed purple-red, purple-black phenotypes, and accumulated more anthocyanin in the leaves, stems and flowers compared with wild type. The results showed that ectopic expression of *EsMYB90* in stems, leaves and flowers of transgenic tobacco could significantly enhanced the expression of anthocyanin biosynthetic genes including EBGs (*PAL*, *CHS*, and *CHI*) and LBGs (*DFR*, *ANS*, and *UFGT*), particularly in LBGs. The study suggested that *EsMYB90* plays a key role in regulating anthocyanin biosynthesis, and it provide new clues to increase the content of anthocyanin in transgenic plants.

## Methods

### Plant materials and growth conditions

The seeds of *Eutrema salsugineum* (Shandong ecotype), *Arabidopsis thaliana* (Columbia-0) and tobacco (*Nicotiana tabacum* cv SR1) are preserved and presented by Shandong Provincial Key Laboratory of Plant Stress Research, College of Life Science, Shandong Normal University.

Wild-type and transgenic plants of *Nicotiana tabacum* and *Arabidopsis thaliana* (Columbia-0) were grown in a mixture of vermiculite, perlite and peat moss (1:1:1) in a greenhouse with 25 °C, a photoperiod of 16 h light /8 h dark. *Eutrema salsugineum* were grown in a growth chamber with 22 °C, a photoperiod of 16 h light /8 h dark and 70% relative humidity.

T3 generation homozygous transgenic and wild-type tobacco plants were used for analyses. After 8 weeks growth of tobacco plants at the period of 8–9 leaves, the sixth leaves of wild-type and transgenic plants were collected for transcriptome and qRT-PCR identification of the transcriptome. Three biological repeats for transcriptome and qRT-PCR were performed. In tobacco, stems, young leaves (YL), mature leaves (ML), flowers, and fruit pods at the flowering stage, and mature seeds were used to determine the content of anthocyanins, meanwhile the stems, young leaves (YL), and flowers were used to examine expression of anthocyanin biosynthesis genes. Similarly, *Arabidopsis* roots, stems, leaves, flowers, and fruit pods from plants growing 4 weeks, and mature seeds were used to determine the content of anthocyanins. In all cases, samples were frozen immediately in liquid nitrogen and stored at − 80 °C. Three repeats of all tests were conducted.

### Phylogenetic analysis

A set of associated MYB protein sequences were downloaded from the NCBI and multiple sequence alignments were carried out using the DNAMAN software (Version 5.2.2). The phylogenetic tree was constructed using the MEGA6 software with the neighbor-joining method.

### Subcellular localization of *Es*MYB90

The coding region of *EsMYB90* was PCR-amplified by YFPMYB-F and YFPMYB-R primers with *Sac*I and *Sac*II sites, respectively (Additional file 9) and then cloned in to the YFP-pCAT vector, resulting in the *35S:YFP-EsMYB90-pCAT* vector. The onion epidermal cells were transformed with the *35S:YFP-EsMYB90-pCAT* and *YFP-pCAT* (control) plasmids, respectively, using the plasmid bombardment method [61]. The transformed cells were incubated at 25 °C for 16–24 h, then the florescence signals were observed and recorded with a fluorescence microscope (BX51, model 7.3, Olympus, Tokyo, Japan). At least three replicates for each construct were performed.

### RNA isolation and cDNA synthesis

Total RNAs were isolated from 0.5 g of young leaves of *Eutrema salsugineum* and *Nicotiana tabacum* using the TRIzol Reagent (Life Technologies) or Quick RNA isolation kit (Biotech biotechnology company, Beijing, China). The RNA concentration and purity(A260/A280) were measured with a NanoDrop ND1000 spectrophotometer (NanoDrop Technologies, Wilmington, DE, USA). First-strand cDNA was synthesized using the Rever Tra Ace qPCR RT Master Mix with gDNA Remover (Toyobo, Japan). Briefly, RNA was heat-denatured at 65 °C for 5 min and immediately placed on the ice for cooling, then 2 μL of 4 × DN Master Mix, 0.5 pg-0.5 μg of RNA template and nuclease-free water were added to 8 μL of volume. After 5 min of incubation at 37°, 5 × RT Mater Mix II was added to make 10 μL of final volume. RT reaction was carried out at 37 °C for 15 min, 50 °C for 5 min, 98 °C for 5 min to obtain 10 μl of cDNA.

### Construction generation and plant transformation

The whole coding sequence of *EsMYB90* was PCR-amplified from the *Eutrema salsugineum* cDNA using the forward primer 5′-CCGGAATTCTTTAGAATACTTATTGGTCC-3′ and the reverse primer 5′-CGCGGATCCATCAGAGACAGATATTAGTTGG-3′ with *Eco*R I and *Bam*H I restriction enzyme sites at the 5′ and 3′, respectively (Additional file 9). The resulting *EsMYB90* fragment was cloned into the pMD18-T vector (Takara, USA). After sequencing confirmation, the *EsMYB90* fragment was subcloned into the *Eco*RI - *Bam*HI sites of the pCAMBIA3301H vector, where *EsMYB90* was under the control of the *CaMV 35S* promoter. The expression vector (*35S*:*EsMYB90-pCAMBIA3301H*) was finally introduced into the *Agrobacterium tumefaciens* strain GV3101.

Transformation of *N. tabacum* was performed using the leaf disc method essentially as reported by Horsch et al. [62]. Transgenic tobacco seedlings were selected on the MS medium containing 6 mg / L of bastar and 300 mg / L of cefalexin. Transformation of *A. thaliana* Columbia-0 was performed using the floral-dipping method [63] and transformants were screened by spraying 0.1% of bastar herbicide. The presence of the transgene was further confirmed by PCR using specific primers for *EsMYB90*. The homozygous transgenic *N. tabacum* and *A. thaliana* were used for subsequent phenotypic and functional analysis.

### Anthocyanin analysis

Stems, young leaves (YL), mature leaves (ML), flowers, and fruit pods of tobacco growing about 8 weeks at the flowering stage, and the mature seeds were sampled, respectively. Similarly, stems, leaves, flowers, fruit pods, and roots of *Arabidopsis* growing about 4 weeks at the bolting period, as well as the mature seeds were also collected. All materials were frozen immediately in liquid nitrogen and ground to powders. The anthocyanin content was determined using an improved method described by Neff and Chory [64]. The measurements of *A*_530_ and *A*_657_ were conducted with a spectrophotometer (UV-1800, Shimadzu). The results were calculated by the eq. (A530–0.25*A657)/fresh weight. Three replicates were performed for each sample.

### RNA-seq and bioinformatic analysis

Total RNAs from the sixth leaves of wild-type and *EsMYB90* transgenic tobacco at the 7–8 leaves stage were isolated using a Quick RNA isolation kit (Bioteke Corporation, Beijing, China). The RNA library construction and sequencing were performed in the BGI Corporation (Shenzhen, China) using the BGISEQ-500 platform. Three independent biological replicates were carried out.

The low-quality reads (more than 20% of the bases qualities are lower than 10), reads with adaptors and reads with unknown bases (N bases more than 5%) were filtered to get the clean reads. The clean reads were mapped to the reference genome using HISAT [65]. Meanwhile, the clean reads were mapped to the reference transcripts using Bowtie2 [66]. The clean reads were assembled into unigenes, followed by the unigene functional annotation, etc., and calculate the unigene expression levels of each sample [67]. Finally, we identified DEGs (differential expressed genes) and performed clustering analysis and functional annotations. DEGs with the GO and KEGG annotation results were classified according to the official classification, and the GO and KEGG pathway functional enrichment were performed using phyper in the R software. Transcription Factor Prediction of DEG: The ORF of each DEG were founded using getorf and aligned to TF domains (from PlntfDB) using hmmsearch [68].

### Gene expression analysis by qRT-PCR

To validate the transcriptome results, the real-time qPCR was performed by the LightCycler® 96 system to examine expressions of selected genes (Additional file 9) using the total RNAs extracted from tobacco leaves used for RNA-seq. Transcriptome data was verified by comparing the result of qRT-PCR (−ΔΔCT) with RNA-seq (log2FC).

To examine expression of anthocyanin biosynthesis genes in different tissues, stems, young leaves (YL) and flowers of tobacco at the flowering stage were sampled, respectively. The total RNAs were extracted using the total RNA rapid extraction kit (Biotech biotechnology company, China). The first-strand cDNAs were synthesized using the Rever Tra Ace qPCR RT Master Mix with gDNA Remover (Toyobo, Japan). The qPCR was performed using the LightCycler® 96 system (Roche, Switzerland; Supplementary Table S1). *Ntubi2* (*ubiquitin 2*, LOC107772211) was used as an internal reference gene. Three replicates were performed for each sample.

## Supplementary information

**Additional file 1. **Fig. 1. Molecular identification of *EsMYB90* transgenic tobacco and *Arabidopsis* plants. Fig. 2. Pathways of the most enriched KEGG DEGs in RNA-Seq of *EsMYB90* transgenic tobacco. Fig. 3. Sequence alignment analysis of the EsMYB90 and other EsMYB proteins in *E.salsugineum*.

**Additional file 2. **Differentially expressed genes (DEGs) that meet log2 Fold Change ≥1 or ≤ − 1 and a Padj ≤0.05 in RNA-Seq of *EsMYB90* transgenic tobacco.

**Additional file 3. **Annotated DEGs in GO terms in RNA-Seq of *EsMYB90* transgenic tobacco.

**Additional file 4. **Annotated DEGs in KEGG in RNA-Seq of *EsMYB90* transgenic tobacco.

**Additional file 5.** Top 20 paths according to the RichFactor value of DEGs.

**Additional file 6.** DEGs enriched in the flavonoid and anthocyanin synthesis pathways.

**Additional file 7.** Detailed data for validation of RNA-Seq results using quantitative real-time PCR.

**Additional file 8. **Detailed data for the expression analysis of anthocyanin biosynthesis genes in stem, young leaf and flower from two *EsMYB90* transgenic tobacco lines and WT.

**Additional file 9.** Sequences of the primers used in the study.

## Data Availability

All data generated or analyzed during this study are included in this published article and its additional files. The raw data of RNA library were available at NCBI Short Read Archive (PRJNA609528).

## Abbreviations

PAs: Proanthocyanidins
PAL: Phenylalanine ammonia-lyase
CHS: Chalcone synthase
CHI: Chalcone isomerase
DHK: Dihydrokaempferol\
F3H: Flavanone 3 β-hydroxylase
DHQ: Dihydroquercetin
F3’H: Flavonoid 3’-hydroxylase
DHM: Dihydromyricetin
F3’5’H: Flavonoid 3’,5’-hydroxylase.
DFR: Dihydroflavonol 4-reductase
ANS: Anthocyanidin synthase
UFGT: UDP-glucose flavonoid 3-O-glucosyltranferase
MBW: MYB-bHLH-WD40
EBGs: Early biosynthesis genes
LBGs: Late biosynthesis genes
YL: Young leaves
ML: Mature leaves
DEGs: Differentially expressed genes
KEGG: Kyoto Encyclopedia of Genes and Genomes
GO: Gene Ontoloty
qRT-PCR: Quantitative reverse transcription PCR
