# Supplementary material for: Identification of the Eutrema salsugineum EsMYB90 gene important for anthocyanin biosynthesis
Source: BMC Plant Biol. 2020 Apr 28;20:186. doi: 10.1186/s12870-020-02391-7 (PMC7189703; doi:10.1186/s12870-020-02391-7)
Supplement: Supplementary file 1 — Additional file 1. Fig. 1. Molecular identification of EsMYB90 transgenic tobacco and Arabidopsis plants. Fig. 2. Pathways of the most enriched KEGG DEGs in RNA-Seq of EsMYB90 transgenic tobacco. Fig. 3. Sequence alignment analysis of the EsMYB90 and other EsMYB proteins in E.salsugineum. [file 12870_2020_2391_MOESM1_ESM.pdf]

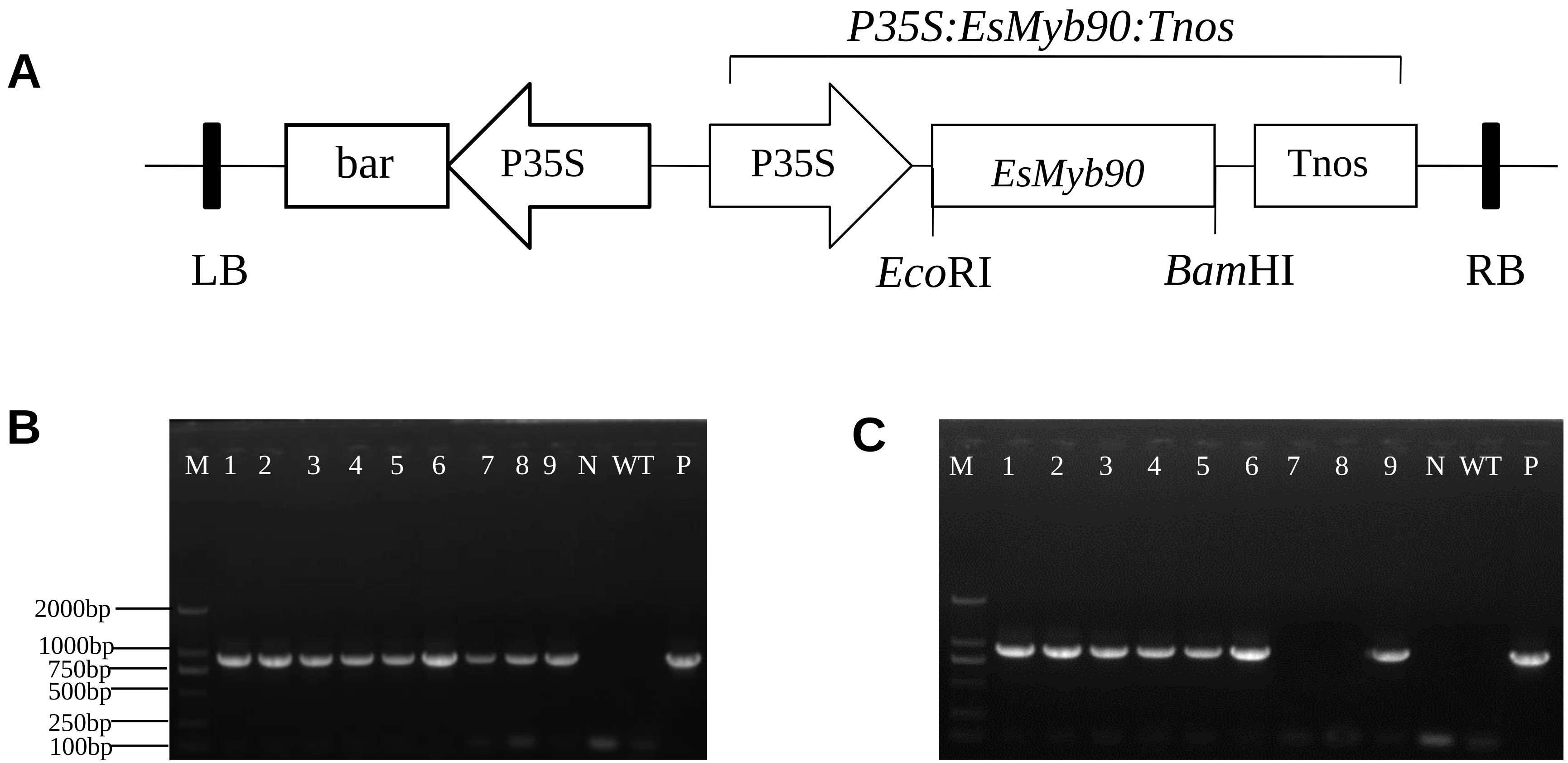

Additional file 1: Fig.1 Molecular identification of the *EsMYB90* transgenic tobacco and *Arabidopsis* plants.

Additional file 1: Fig.1 Molecular identification of *EsMYB90* transgenic tobacco and *Arabidopsis* plants.

(A) Expression cassette of *pCAMBIA3301H-35S:EsMyb90* overexpression vector

(B) PCR amplification showing the *EsMYB90*'s presence in independent transgenic tobacco lines. 1-9, T1 transgenic tobacco seedlings with herbicide resistance ; N, No template（ddH_2O）; WT, Wild type; P, Positive *pCAMBIA3301H-35S:EsMyb90* plasmid, M: DL2000 DNA marker. The expected amplicon sizes are shown in Additional file 1: Fig. 1B.

(C) PCR amplification showing the *EsMYB90*'s presence in independent transgenic *Arabidopsis* lines. 1-9, T1 transgenic *Arabidopsis* seedlings with herbicide resistance ; N, No template（ddH_2O）; WT, Wild type; P, Positive *pCAMBIA3301H-35S:EsMyb90* plasmid controls, M: DL2000 DNA marker. The expected amplicon sizes are shown in Additional file 1: Fig. 1C.

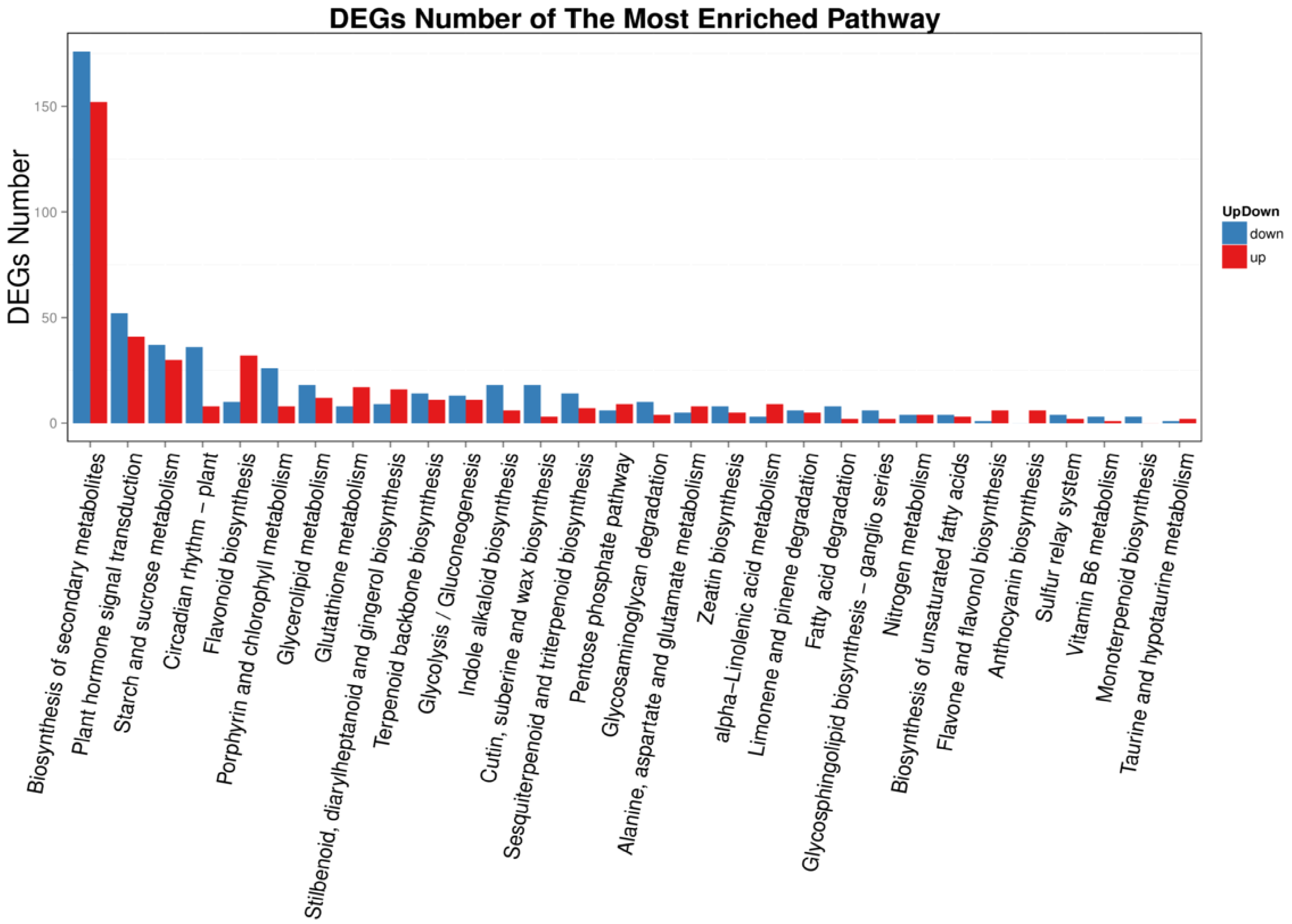

Additional file 1: Fig.2 Pathways of the most enriched KEGG DEGs in RNA-Seq of *EsMYB90* transgenic tobacco. The X axis represents the pathway entry. The Y axis indicates the number of up and down regulated genes for the corresponding pathways.

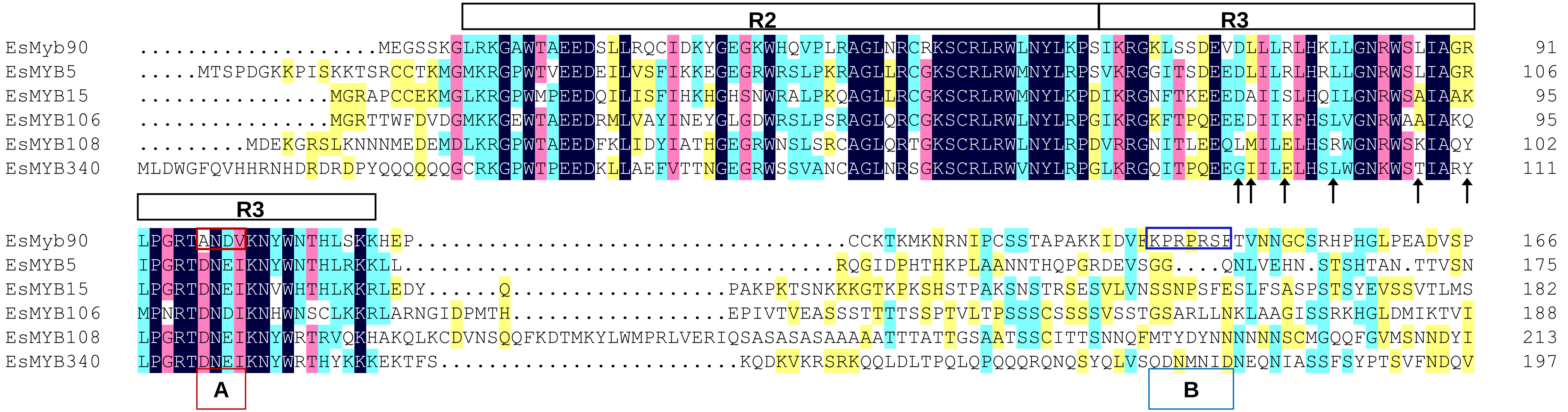

Additional file 1: Fig.3 Sequence alignment analysis of the EsMYB90 and other EsMYB proteins in *E.salsugineum* .

Additional file 1: Fig.3 Sequence alignment analysis of the EsMYB90 and other EsMYB proteins in *E.salsugineum*. EsMYB90 protein sequence was aligned with five EsMYBs [Myb5(XP_006407201),Myb15(XP_006406061),Myb106(XP_006390428),Myb108(XP_006407939),Myb-related Protein 340(XP_006395259)] from *E.salsugineum*. Identical amino acids are shaded in dark blue, and the greater than or equal to the identity of 75%, 50% and 33% are indicated in the shades of pink, light blue and yellow, respectively. The R2 and R3 domains refer to two repeats of the MYB DNA binding domain. Box (A): a conserved motif ANDV in the R2R3 domain for dicot anthocyanin-promoting MYBs; Box (B): a C-terminal-conserved motif KPRPR [S/T]F for anthocyanin-promoting MYBs; Black arrows:the specific residues of [D/E]Lx_2[R/K]x_3Lx_6Lx_3R that confer to the interaction with bHLH.
