# Supplementary material for: Identification of the Eutrema salsugineum EsMYB90 gene important for anthocyanin biosynthesis
Source: BMC Plant Biol. 2020 Apr 28;20:186. doi: 10.1186/s12870-020-02391-7 (PMC7189703; doi:10.1186/s12870-020-02391-7)
Supplement: Supplementary file 9 — Additional file 9. Sequences of the primers used in the study. [file 12870_2020_2391_MOESM9_ESM.docx]

**Additional file 9** Sequences of the primers used in the study

| **Gene ID (Accession number)** | **Primer Name** | | | **Sequence of primers（5’-3’）** | **Function of primers** | |
| --- | --- | --- | --- | --- | --- | --- |
| 18008560 | EsMyb90-F | | | CCGGAATTCTTTAGAATACTTATTGGTCC | Construction of *EsMyb90* overexpression vector | |
|  | EsMyb90-R | | | CGCGGATCCATCAGAGACAGATATTAGTTGG |  |  |
| 18008560 | YFPMyb90-F | | | ATGCGAGCTCTAATGGAGGGCTCGTCCAAAG | Construction of *YFP*-*EsMyb90* overexpression vector | |
|  | YFPMyb90-R | | | TATACCGCGGCTAATCAAGTTCCACAGTGTCCC |  |  |
| 107803097 | NtDFR-F | | | ACCTAGCTTAATCACTGCCCTTTC | Quantitative PCR used to validate the transcriptome | |
|  | NtDFR-R | | | TGAATCTCCCCTCTGCCTTTG |  |  |
| 107778118 | NtLODX54-F | | | CAGAACTAGCACTTGGCGTTG |  |  |
|  | NtLODX54-R | | | GCATGATTATGGAATTAGGCACAC |  |  |
| 107781346 | Nt3GT12-F | | | GAAAGTTATGAAAGAGGCAGAG |  |  |
|  | Nt3GT12-R | | | ACCAGAACCAGCAGTCCA |  |  |
| 107781522 | Nt3GT36-F | | | GAACTGGCTTTAGGTCTGGAGC |  |  |
|  | Nt3GT36-R | | | CTGTTGTTGCACCCAACCTG |  |  |
| 107831042 | Nt3GT53-F | | | GAGCAAACTGGGCTACCT |  |  |
|  | Nt3GT53-R | | | CACCCAACCTGAATGAATAA |  |  |
| 107806960 | NtCu-ZnSOD-F | | | TCTCAATCGCTGCCCCTAACAC |  |  |
|  | NtCu-ZnSOD-R | | | AGCAGTGGCAGCAGAAAGTGT |  |  |
| 107772738 | NtP450-F | | | TAATGCTTGGGCAATCGGA |  |  |
|  | NtP450-R | | | TGCCTGCACCAAAGGGAAT |  |  |
| 107827231 | NtPOD44-1-F | | | CAACGCGGGATGCAGTAG |  |  |
|  | NtPOD44-1-R | | | AGCCCTTTGTTTGTGAACGATT |  |  |
| 107797651 | NtPOD44-2-F | | | GCTGGACCAAATCAAACA |  |  |
|  | NtPOD44-2-R | | | TGGAATGGCATAGGTAGG |  |  |
| 107795590 | NtbZIP-F | | | GACGAGGTAGCGGCTGTTGTTG |  |  |
|  | NtbZIP-R | | | TCTGCCGATTATTTCTCCTGAT |  |  |
| 107795213 | NtMyb3R-1-F | | | TCTGCCGATTATTTCTCCTGAT |  |  |
|  | NtMyb3R-1-R | | | GTCTCAGCACCAAATCCA |  |  |
| 107802984 | NtMyb4-F | | | AGAGGTCCGTGGAGCAAAG |  |  |
|  | NtMyb4-R | | | CTCTTAATATCAGGTCGCAAAT |  |  |
| 107825953 | NtWRKY53-F | | | CTCTTAATATCAGGTCGCAAAT |  |  |
|  | NtWRKY53-R | | | ACTAGAGAACGTTGGAGGAAAG |  |  |
| 107761230 | NtAKT2/3-F | | | ATGGCGAGTATGACAAAT |  |  |
|  | NtAKT2/3-R | | | ACCTAAAGGAGGGAGAAT |  |  |
| 107805986 | NtAX15A-F | | | ACCTAAAGGAGGGAGAAT |  |  |
|  | NtAX15A-R | | | ATTGTGACACCACCCATAGGAT |  |  |
| 107803626 | NtCaM1-F | | | TGTAAAGAAAACGCCAAGCATGTCG |  |  |
|  | NtCaM1-R | | | CCACTTCCCATCATCTTC |  |  |
| 107782983 | NtMAPK3-F | | | ATGGATTGGGTTCGTGGTG |  |  |
|  | NtMAPK3-R | | | TGATGAGGGTAGCCGACTGAG |  |  |
| 107806359 | NtMAPK6-F | | | CTTTCACGACGGCGATTTAC |  |  |
|  | NtMAPK6-R | | | ACACCACCACTTCCCTTTCC |  |  |
| 107802063 | | NtPAL-F | ATGGCACTGCTGTTGGTTCTG | | | Quantitative PCR for gene expression analysis |
|  |  | NtPAL-R | CAACTTGTGAGTCAAGTGGTCTGTG | | |  |
| 107826422 | | NtCHS-F | ATCACTGCGGTCACATTTCGT | | |  |
|  |  | NtCHS-R | GAAGAGACAAGCTCGAACAAAGG | | |  |
| 107779699 | | NtCHI-F | TGTTGGGGCATTGACGATTAG | | |  |
|  |  | NtCHI-R | TCCCATCTACTTCTGGCATTG | | |  |
| 107770893 | | NtF3H-F | GACAACCCTTGCTAAACAATTCTTC | | |  |
|  |  | NtF3H-R | TCTTGGACCACTTCACCCTGTAG | | |  |
| 107795677 | | NtF3’H-F | ACGTCCTCAAGCACAGTAGAATG | | |  |
|  |  | NtF3’H-R | TAAGTTAATTGGGCTAGGTCGG | | |  |
| 107803097 | | NtDFR-F | ACCTAGCTTAATCACTGCCCTTTC | | |  |
|  |  | NtDFR-R | TGAATCTCCCCTCTGCCTTTG | | |  |
| 107819370 | | NtANS-F | CCCCTACAGACTACATTCCAGC | | |  |
|  |  | NtANS-R | CCTCCATGCCTCCGACTTC | | |  |
| 107781346 | | NtUFGT-F | AAAATAGTTTCTTGGGCACCTC | | |  |
|  |  | NtUFGT-R | TTCCACCATTCTACGATTCAGC | | |  |
